# Gradient Ion Beams Regulate Surface Group Modification to Enhance Interfacial Charge Transport in Triboelectric Polymers

**DOI:** 10.1002/advs.202518257

**Published:** 2026-01-21

**Authors:** Yi Chen, Yuliang Yao, Xinya Shen, Meiling Gong, Chuan Xu, Chunliang Zhou, Fuqiu Ma, Jian Zhang, Xiangyu Chen, Yanxia Liang, Engang Fu, Yong Fan

**Affiliations:** ^1^ Yantai Research Institute Harbin Engineering University Yantai China; ^2^ State Key Laboratory of Nuclear Physics and Technology School of Physics Peking University Beijing China; ^3^ College of Materials Science and Chemical Engineering Harbin Engineering University Harbin China; ^4^ College of Energy Xiamen University Xiamen China; ^5^ CAS Center for Excellence in Nanoscience Beijing Key Laboratory of Micro‐nano Energy and Sensor Beijing Institute of Nanoenergy and Nanosystems Chinese Academy of Sciences Beijing China; ^6^ State Key Laboratory of Chemistry for NBC Hazards Protection Beijing China

**Keywords:** energy bandgap regulation, flexible triboelectric polymers, ion implantation, polarity enhancement, surface molecular modification

## Abstract

Triboelectric nanogenerators (TENGs) exhibit high sensitivity and flexibility, enabling them to rapidly and effectively respond to high‐entropy mechanical energy sources like friction and contact into utilizable electrical power, TENGs are emerging as one of the most promising devices for future applications in wearable devices and self‐powered sensors. However, the flexible polymers charging materials used in TENGs inherently suffer from low surface charge density, which significantly constrains their electrical performance. This study proposed a gradient ion beam irradiation strategy to engineer functional groups on polymer surfaces through precise dose‐control irradiation, thereby enhancing interfacial charge transport capability. Electrical testing revealed 13‐, 10‐, and 7‐fold improvements in output voltage, current, and surface charge density, respectively, with stability exceeding 1440‐fold that of pure charge injection. Chemical structural evolution under varying irradiation doses was systematically investigated to probe molecular‐scale regulatory mechanisms. Combining density functional theory (DFT) calculations, we found that the energy bandgap of the surface molecular structure decreased after irradiation, and the distribution of the electrostatic surface potential (ESP) indicated an increase in electron energy, thereby elucidating the mechanism underlying the enhanced surface charge transport in charged polymers. Meanwhile, after being fabricated into micro‐devices, they exhibit high sensitivity and excellent abrasion resistance, establishing a theoretical foundation for advancing TENG functionality in wearable sensors and flexible electronics.

## Graphical abstract

This study addresses the core scientific question of atomic‐scale structural units and their assembly mechanisms by integrating ion implantation technology— originally developed in nuclear physics research—with flexible intelligent polymers. Through this interdisciplinary approach, we have enabled on‐demand customization of surface functionalities and interfacial structures in polymer‐based flexible materials, thus establishing a novel strategy for atomic‐level manufacturing. Altogether, this ion implantation–based strategy offers a new pathway for the fabrication of high‐performance wearable sensors and advanced flexible functional materials.

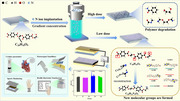

## 1 Introduction

The rapid advancement of intelligent systems and the Internet of Things (IoT) has driven the continuous integration of numerous distributed intelligent devices, particularly wearable and flexible electronic devices, into this expansive network [1, 2, 3]. As the core functional module of intelligent systems, the perception layer relies on decentralized sensors for data acquisition and transmission, significantly impacting the overall system intelligence [4, 5, 6]. While most small wireless sensors operate at low power levels (milliwatts to watts), those deployed in complex environments encounter power supply challenges. Conventional power systems often fail to deliver accurate and continuous energy in such settings, leading to potential energy waste and hindering the long‐term autonomous operation required by distributed intelligent sensors [7, 8]. Harvesting low‐power, high‐entropy ambient energy sources (e.g., wind energy) to develop self‐powered sensor systems thus presents a viable and practical solution for meeting the energy demands of distributed applications. Consequently, technologies such as piezoelectric nanogenerators (PENGs), triboelectric nanogenerators (TENGs), and magnetoelastic generators (MEGs), have been developed specifically for mechanical energy harvesting [9, 10].

The triboelectric nanogenerator (TENG), pioneered by Wang et al., operates through the synergistic mechanisms of contact electrification and electrostatic induction [11]. Demonstrating its practical utility, TENG tactile sensors fabricated from Ecoflex 00–30 silicone polymer have replaced conventional, less effective, and higher‐cost piezoelectric sensors in a humanoid mechanical hand. These TENG tactile sensors significantly enhance shape and material recognition accuracy, achieving 98% and 99% accuracy, respectively [12]. Owing to exceptional energy conversion efficiency, structural diversity, and operational flexibility, TENG technology has established itself as a cornerstone for micro‐nano energy systems, exhibiting outstanding potential for distributed deployment across diverse applications [13, 14].

The properties of triboelectric material critically influence triboelectric charging and charge transfer behavior, fundamentally determining the output performance of the TENG. Conventional triboelectric materials, such as PET and PTFE (polytetrafluoroethylene), exhibit intrinsic limitations, including low charge density, inadequate dielectric properties, and insufficient environmental stability, which constrain further performance enhancements [15, 16, 17]. Beyond charging material selection, surface modification has emerged as an effective strategy to enhance charge density and improve TENG performance [18, 19, 20].

Recent research has increasingly focused on ion migration and ion cross‐linking plasma‐based modification techniques, supplementing traditional molecular‐level physical and chemical modifications [21, 22, 23]. These plasma‐based methods offer precise control, superior performance, and environmental compatibility while overcoming limitations of conventional modifications [24, 25]. Ion mobility modification enhances ion migration rate by constructing continuous ion transport channels using conductive materials (e.g., polypyrrole) or ion carriers (e.g., carbon nanotubes) [26]. For instance, Liu et al. successfully fabricated conductive PPy@ChNCs composite from chitin nanocrystals (ChNCs) and polypyrrole (PPy), deploying it as the liquid electrode in single‐electrode triboelectric nanogenerators (PC‐TENGs). This material exhibited a threefold increase in electrical conductivity, leading to a 71% enhancement in the TENG's output voltage [27]. However, the synthesis involves complex multi‐step polymerization and precise dispersion techniques, resulting in high processing complexity and limited stability. Furthermore, excessive reliance on the conductive network compromises material stiffness and reduces mechanical strength.

Ion crosslinking establishes reversible ion transport channels by combining dynamic bonds and other strategies with polar groups (e.g., hydroxyl, carboxyl) in polymer chains. This forms an ion bond network that modulates the dielectric response and enhances material properties [28]. Zheng et al., for example, substantially improved the mechanical properties by incorporating β‐lactoglobulin precursor fibers into PVA polymers and chemically cross‐linking them with BF via hydrogen bonds, thereby forming a stable PVA aerogel network. Compared to pure PVA porous films, this achieved an eightfold increase in breaking strength and a fourfold improvement in flexibility [29]. However, prolonged cycling of BF‐PVA can lead to disulfide bond degradation, impacting its cycling lifespan.

Both ion migration modification and ion cross‐linking approaches encounter challenges, including intricate procedures, inadequate stability, and insufficient mechanical strength, hindering their widespread application. Consequently, investigating novel surface modification strategies remains imperative for improving the electrification performance of polymer materials.

Ion irradiation technology has emerged as an efficient method for material surface modification, attracting growing interest across various research fields [30, 31]. Through the interaction between high‐energy ion beams and the material surface, this technique induces chemical bond breakage and reconstruction, generates polar groups (e.g., ‐CN), modulates surface charge distribution, and precisely tailors physical‐chemical properties. This process imparts novel functional characteristics to materials [32]. For instance, Fu et al. achieved electrical property reversal in polyimide (Kapton) through helium ion irradiation, attaining a high charge density of 332 µC/m^2^. This significantly enhanced charge transport capacity while maintaining its flexibility and insulation properties [33]. Nitrogen ion beam (N^+^), with high electronegativity (χ = 3.04) and significant chemical reactivity, enables cost‐effective enhancement of flexible polymers’ electrical properties. The implanted C≡N groups exhibit robust electron‐withdrawing capability, endowing polymers with electronegativity surpassing fluorinated analogues at merely one‐third of their modification cost. This renders nitrogen ion implantation an ideal strategy for high‐performance flexible electronics. Furthermore, nitrogen ion implantation in flexible polymers like PTFE and FEP (fluorinated ethylene propylene) enhances surface charge density by 4–8 times (reaching up to 230 µC/m^2^) and increases peak power density by a factor of a thousand [34]. Nevertheless, the precise regulation of ion implantation effects and the underlying mechanisms for enhancing surface charge transport in flexible polymer charging materials remain insufficiently elucidated.

This study proposes a novel approach utilizing a gradient nitrogen ion beam irradiation system to modify flexible PET polymer materials. By precisely controlling the irradiation dose, this technique facilitates targeted chemical bond reconstruction and tailored design of polar groups. This strategy effectively overcomes the limitations imposed by PET's chemical inertness, transforming it into a high‐performance charging material. Multiscale analysis spanning electronic and molecular structural levels reveals ion implantation regulatory pathways and the charge transport enhancement mechanisms. These findings establish a material foundation for advancing TENG applications in wearable sensing and micro/nano energy systems.

## 2 Materials and Methods

### 2.1 Reagents and Chemicals

Sample preparation: Transparent PET polymer sheets (thickness: 100 µm, density: 1.37 g/cm^3^) were cut into 1 cm × 1 cm specimens. These samples were ultrasonically cleaned sequentially in methanol and acetone baths for 10 min each to remove surface contaminants. Following cleaning, samples were rinsed thoroughly with deionized water, dried under a stream of nitrogen gas, and stored in a vacuum desiccator. The prepared PET polymer samples were fixed onto the platform of the ion implanter. N‐ion beam irradiation was performed at a fixed energy of 100 keV. Subsequently, the samples were irradiated at three different fluences: 5 × 10^14^ ions/cm^2^ (designated PET‐5E14), 1 × 10^15^ ions/cm^2^ (PET‐1E15), and 1 × 10^16^ ions/cm^2^ (PET‐1E16).

### 2.2 Flexible Polymers Characterization and Analysis

We systematically investigated chemical structure changes in ion‐implanted polymer samples. The feasibility of the ion implantation was validated through the stopping and range of ions in matter (SRIM) simulations. Density functional theory (DFT) calculations were performed to simulate and analyze energy bandgap alterations in PET samples before and after irradiation, providing mechanistic insights. The chemical composition and bonding states were characterized using X‐ray photoelectron spectroscopy (XPS) (model). Molecular vibrational modes and structural change were assessed via a Raman spectrometer (model). Functional groups and chemical bonding of samples were examined using attenuated total reflectance‐fourier transform infrared spectroscopy (ATR‐FTIR) (model). The changes in the microstructure of the sample were observed using a scanning electron microscope (SEM). Employ atomic force microscopy (AFM) and conductive atomic force microscopy (C‐AFM) to explore the 3D topography of the sample surface and the underlying mechanism affecting its electrical properties. Additionally, electrical properties were evaluated using a Keithley 6514 system electrometer, a digital linear motor, and an impedance analyzer (model).

## 3 Results and Discussion

### 3.1 Simulation and Mechanism Analysis

**FIGURE 1:**
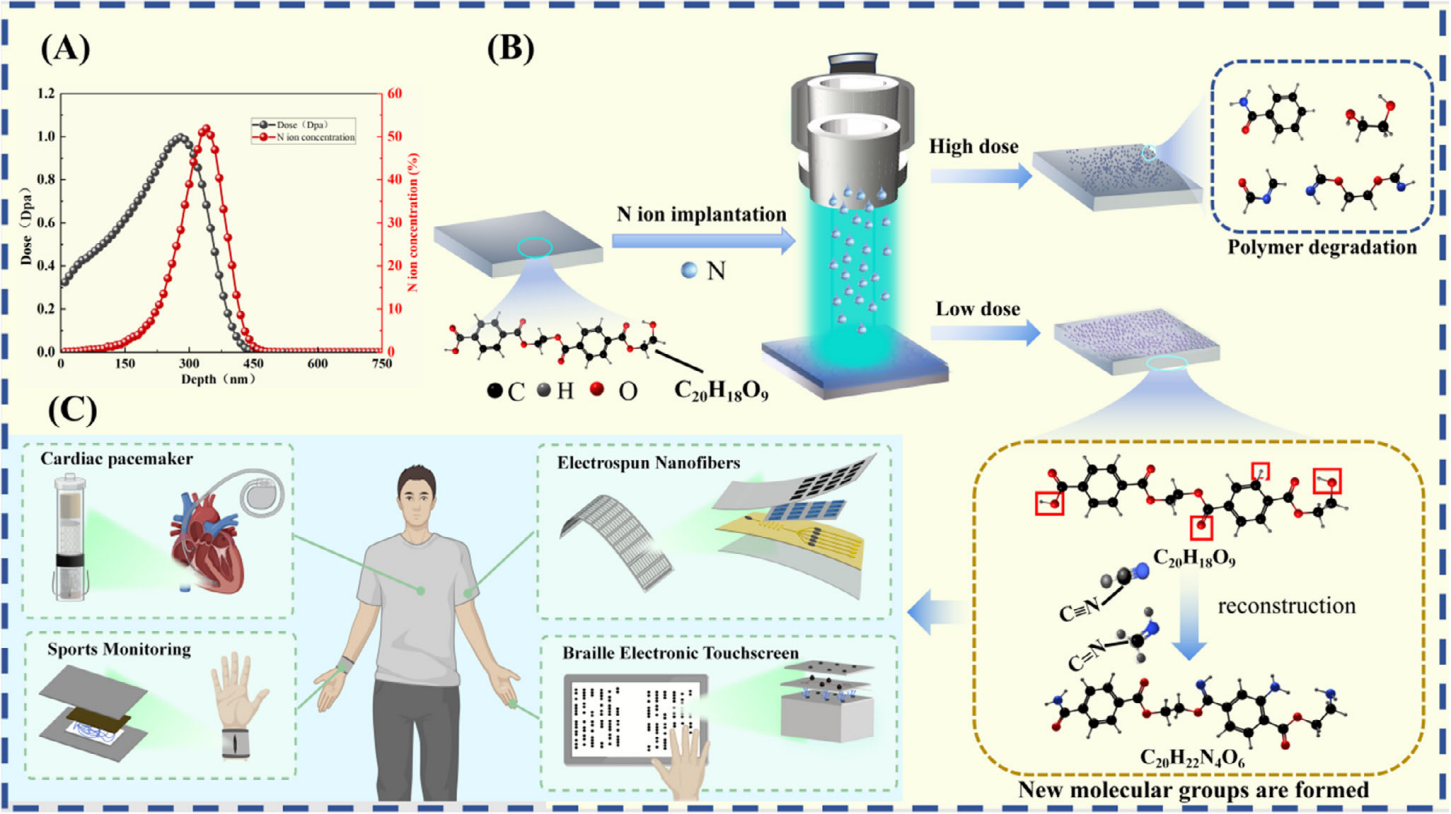
SRIM implantation damage along with N ion concentration distribution as a function of depth (A), 100 keV N ion injection into PET materials, illustrating the possible reaction pathways (B), and the application fields of the modified flexible PET polymer‐based TENG are thoroughly explored (C).

The PET material was implanted with 100 keV N ions. Figure 1B illustrates a potential reaction pathway modulated by implantation dose. Nuclear energy loss during N‐ion implantation induces atomic displacement along the PET molecular chain, cleaving chemical bonds and generating diverse free radicals. Concurrently, incident N ions react with these generated radicals, forming new chemical bonds, demonstrating the ion implantation effect in polymers. Critically, implanted N ions form highly polar groups, which increase the electric dipole moment of PET molecular chains, thereby enhancing molecular polarity. At lower fluence (PET‐5E14), ion implantation effects dominate: surface molecules polarity significantly enhances triboelectric performance versus pristine PET. However, increasing doses intensifies the PET main chain scission and polymer degradation. These two effects coexist at higher doses. At PET‐1E16, substantial molecular chain degradation occurs on the PET surface, potentially causing a significant decline in electrical properties.

To quantify these effects, SRIM software calculated the projected range and irradiation‐induced damage of the implanted N ions. This simulation tracks transport and collision of a large number of incident particles within the material, calculating energy loss of secondary particles and diverse parameters across the irradiation process [35]. When ions strike the material surface, their trajectories exhibit a zigzag pattern within the solid. The trajectory of ion in solid is defined as the total range (R), expressed as follows:

(1) $$R=\int_{0}^{E0}\frac{dE}{-dE/\mathrm{dx}}=\int_{0}^{E0}\frac{dE}{NS\left(E\right)}$$

In the formula, E_0_ denotes the initial energy, (dE/dx) represents the stopping power (energy loss per unit length of energetic ions in the solid), N signifies the atomic number density of the target material, and S(E) denotes the nuclear stopping cross‐section per atom. The distribution of ion trajectories and their concentration is determined by physical parameters, including the ion collision mean free path (l), scattering angle (θr), and velocity azimuth angle (χ) in the experimental coordinate system. Considering that surface charge transfer in the vertical contact‐separation TENG mode primarily occurs within a near‐surface region (<500 nm), SRIM simulations were employed to design an effective modification depth for nitrogen ion implantation not exceeding 450 nm (Figure 1A). The simulated peak damage level reaches 1.03 displacements per atom (dpa), with a peak implanted N ions concentration of 53 at%. Radiation damage (dpa) and concentration of the injected N ion along the depth in PET by N ion irradiation are at various dose levels according to SRIM (Figure S1). After determining the ion implantation depth range through SRIM simulations, we employed ion irradiation with gradient doses to precisely modulate the molecular structure of the sample surface. The control mechanisms governing ion implantation effects under different doses are illustrated in Figure 1B. For low‐dose ion implantation, the primary modification mechanism involves implantation effects: the ion beam energy selectively breaks lower‐energy ester bonds within the molecular chains, while the implanted nitrogen ions participate in forming polar bonds and polar functional groups. This process enhances the material's polarity and improves its triboelectric performance. Conversely, high‐dose ion implantation severely disrupts the molecular chain structure of the sample, causing the sample surface to undergo carbonization. The underlying regulatory mechanisms and charge transport enhancement mechanisms will be further elaborated in subsequent sections.

The modified PET polymer exhibits high electronegativity, making it an ideal triboelectric material for vertical contact‐separation mode TENGs. This characteristic underscores its significant application potential in wearable devices and electronic functional devices, as demonstrated in Figure 1C. Its excellent biocompatibility enables applications in self‐powered systems such as cardiac pacemakers, while its superior flexibility supports health monitoring applications in flexible wearable devices. Furthermore, this flexible polymer can be utilized in Braille electronic touchscreens to provide assistance for visually impaired individuals. Due to its exceptional wear resistance and mechanical stability, this PET polymer is particularly well‐suited for manufacturing array‐structured TENG‐based flexible self‐powered sensors, which can be seamlessly integrated into skin‐attachable electronic devices.

The microscopic electronic structure modifications of the samples were analyzed using density functional theory (DFT) calculations. As a computational quantum mechanical approach, DFT is founded on the Hohenberg–Kohn theorems and implemented through the Kohn–Sham equations. It enables the investigation of many‐electron systems ground state properties and is extensively utilized to simulate the electronic structures of both molecules and solid‐state materials. The central concept of DFT determines a system's physical properties using the electron density distribution function ρ(r), providing an alternative to traditional wave function‐based methods. The ρ(r) is derived from the single‐electron wave function ψ(r) and is mathematically expressed as:

(2) $$\rho \left(r\right)=\sum_{i=1}^{N}{\left|\psi_{i}\left(r\right)\right|}^{2}$$

**FIGURE 2:**
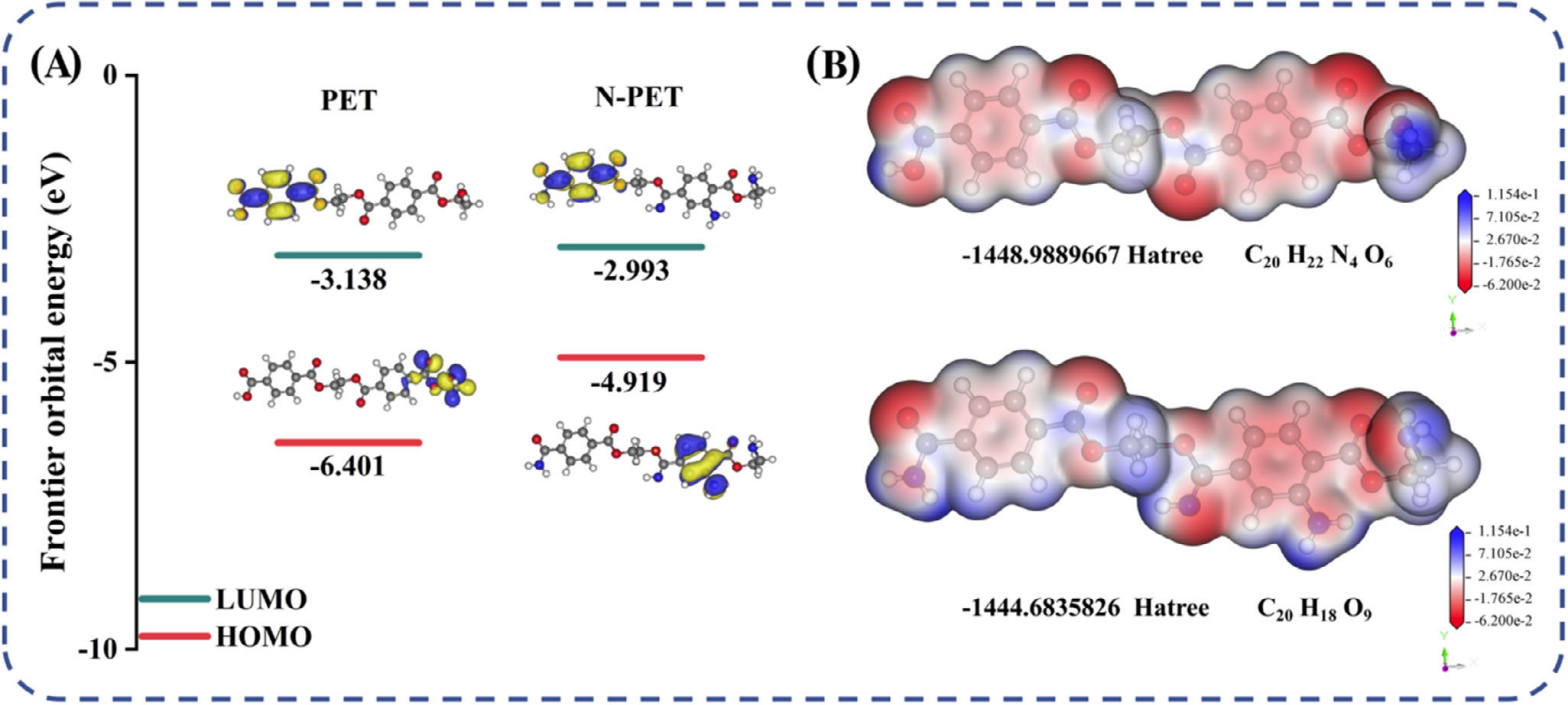
HOMO‐LUMO diagrams illustrating the energy bandgap variations of the PET molecule before and after implantation (A), and surface electrostatic potential distribution diagrams depicting the associated changes (B).

Here, r denotes the spatial coordinates. DFT was employed to calculate the HOMO‐LUMO gap, analyze the electronic band structures, and investigate the surface electrostatic potential (ESP) distributions. To elucidate the mechanism underlying the enhanced surface charge transport properties in polymers, we analyzed the variations in bandgap and surface ESP distribution of the samples before and after irradiation [36]. HOMO‐LUMO calculations for pristine and irradiated PET samples (Figure 2A) revealed a bandgap reduction from 3.263 to 1.926 eV following low‐dose nitrogen ion irradiation. This narrowed bandgap facilitates easier electron excitation into the conduction band (LUMO), enhancing carrier mobility. Concurrently, the LUMO energy level increased from −3.138 to −2.993 eV after irradiation, indicating enhanced electron affinity. These results demonstrate that nitrogen ions incorporation improves the material's electron‐accepting capability.

The ESP distribution of PET molecules reveals that, following irradiation, the electronic energy of the molecular structure on the PET surface increases by approximately 4.3 Hartree (Figure 2B). This energy elevation is attributed to a significant rise in the HOMO level and a concurrent narrowing of the energy bandgap, which is consistent with the computational results presented in Figure 2A. At the same time, a notable reduction in surface electrostatic potential is observed in the irradiated samples, indicating a localized accumulation of negative charge, particularly in regions with high electron cloud density. These low‐potential areas exhibit stronger electrostatic attraction toward positively charged species (e.g., protons and cations), while their repulsion of negative charges becomes more pronounced [37]. This phenomenon arises from the interaction between implanted nitrogen ions and fragmented molecular sites, leading to the reconstruction of molecular chains and the formation of numerous nitrogen‐containing polar covalent bonds and functional groups with high electronegativity. These newly introduced polar structures significantly lower the material's surface potential and increase the dipole moment of the PET molecular chains, thereby enhancing the overall molecular polarity. Collectively, these structural and electronic transformations substantially improve the charge transport capabilities of the PET film, resulting in enhanced electrical performance.

### 3.2 Electrical Performance Analysis

**FIGURE 3:**
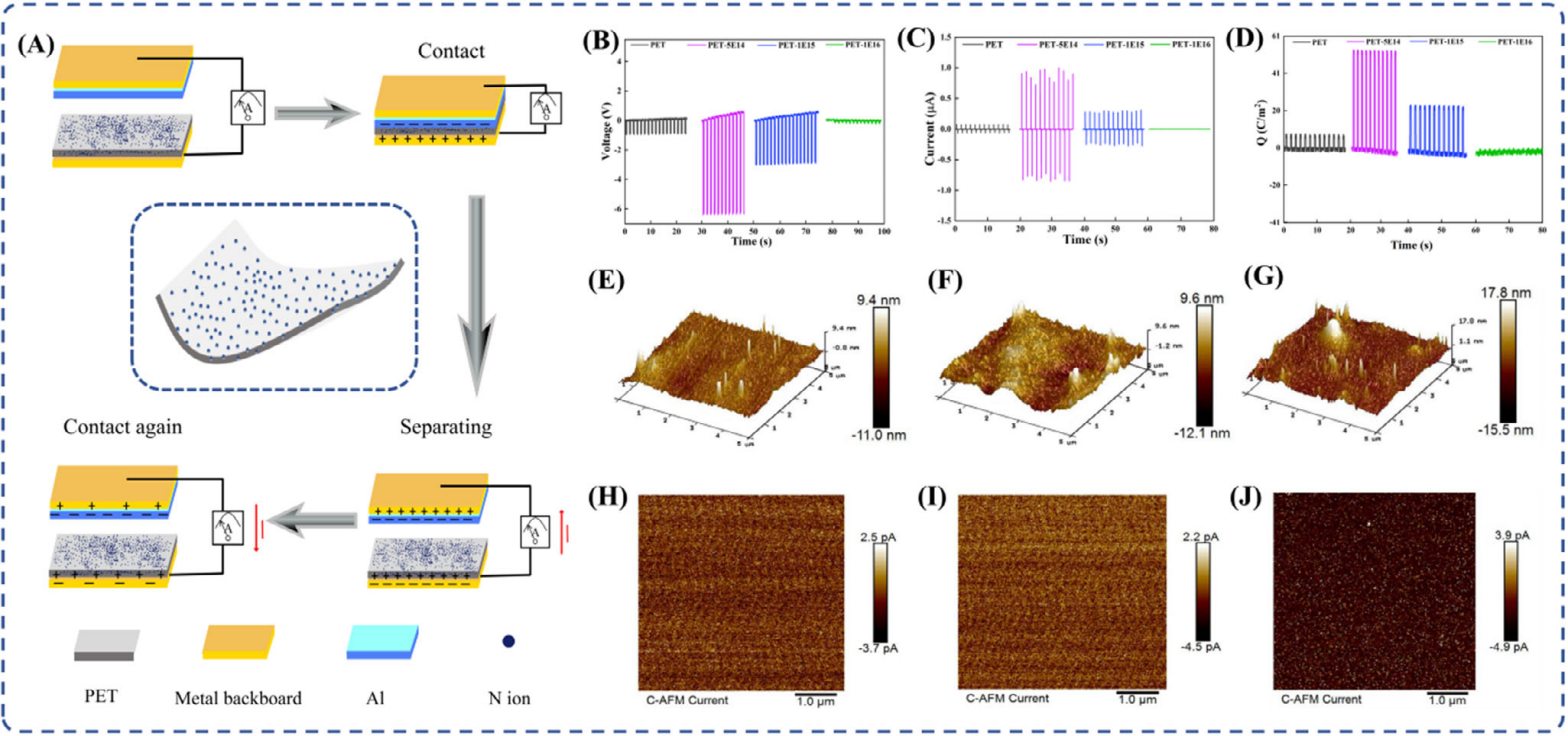
Structure of the TENG and the electrification process during contact separation (A); output voltage (B), output current (C), and surface charge density (D) from the TENG electrical performance test; 3D stereoscopic images (E–G) and local current distribution maps (H–J) of surface topography for PET, PET‐5E14, and PET‐1E16.

The electrical properties of PET materials irradiated with 100 keV nitrogen ions at varying doses were systematically studied. TENG operating in vertical contact‐separation mode was fabricated using irradiated PET samples. Electrical characterization was performed to compare pre‐ and post‐ irradiation performance. This TENG operational mode relies on the principles of contact electrification and electrostatic induction. Its structural configuration and charge transfer dynamics are detailed in Figure 3A. When two triboelectric materials contact, opposite charges are generated at their interface through electron transfer. Upon separation, these friction‐induced charges of opposite polarities remain preserved on their surfaces, thereby forming an electrostatic field between the separated materials. This field drives current flow through connected external circuits. By measuring transferred charge, voltage, and current in the external circuit of TENG devices, the surface charge density of PET under different irradiation doses was determined, enabling analysis of N‐ion induced performance variations. Figure 3B–D respectively present the output voltage, current, and surface charge density of contact‐separated mode TENG devices fabricated by contacting aluminum foil with nitrogen ion‐irradiated PET materials. All devices maintained a consistent 7 mm × 7 mm effective contact area. For unirradiated PET devices, measurements showed 0.5 V output voltage, 0.1 µA current, and 8.16 C/m^2^ surface charge density. Surprisingly, when using PET irradiated with low‐dose of nitrogen ions (5E14), these performance metrics increased significantly—by 13 times (6.5 V), 10 times (1 µA), and 7 times (51 C/m^2^), respectively. This enhancement is attributed to specific defect states introduced by low‐dose N‐ion irradiation, which enhance PET molecular chain polarity and thereby promote the contact electrification effect in TENG devices. However, further increasing the N‐ion irradiation dose progressively reduced the TENG output. At 1 × 10^15^ ions/cm^2^ (1E15), values decreased to 3 V, 0.3 µA, and 26.5 C/m^2^ (still exceeding pristine sample). Critically, at 1 × 10^16^ ions/cm^2^ (1E16), the performance of the TENG devices significantly deteriorated and became nearly undetectable. This decline is attributed to pronounced PET main chain scission and molecular degradation under higher ion doses, which severely damage surface chemical structure and consequently impair electrical performance. As observed through SEM, SEM imaging of the pristine sample reveals a homogeneous, defect‐free surface topography (Figure S2A,B). High‐dose irradiation (1 × 10^16^ ions/cm^2^) induces substantial morphological transformation (Figure S2C,D), with a distinct carbonized layer becomes evident on the sample surface. This carbonization arises from molecular chain fracture caused by high‐dose ion bombardment. Sample edges exhibit foam‐like or lamellar structures, with the overall surface architecture experiencing structural collapse, so these changes ultimately lead to a significant deterioration of electrical performance.

To determine whether the performance degradation of triboelectric nanogenerators (TENGs) under high‐dose irradiation (1E16) is primarily caused by molecular chain scission on the polyethylene terephthalate (PET) surface rather than the formation of conductive leakage pathways, this study employed conductive atomic force microscopy (C‐AFM) to characterize three representative samples: pristine PET, PET‐5E14 (the sample exhibiting optimal performance), and PET‐1E16 (subjected to high‐dose ion irradiation). For each sample, a random 5 µm × 5 µm region on the surface was selected for simultaneous acquisition of topographical and current distribution images. In Figure 3E–G, the surface of the pristine PET sample (Figure 3E) appeared relatively smooth. The PET‐5E14 sample (Figure 3F) displayed nanoscale roughness variations, indicating mild surface damage induced by ion implantation. In contrast, the PET‐1E16 sample (Figure 3G) exhibited significantly increased surface roughness, reflecting more severe structural damage under high‐dose irradiation. The corresponding current mapping results (Figure 3H–J) revealed no detectable current signal across the scanned area of the pristine PET (Figure 3H); any signals below several pA were considered background noise, resulting in an entirely dark image with no bright regions, indicating negligible conductivity. Similarly, the PET‐5E14 sample (Figure 3I) showed extremely low current signals without the presence of any continuous conductive channels, suggesting that while low‐dose modification slightly enhanced the material's electrical behavior, it did not lead to the formation of conductive pathways. In the high‐dose PET‐1E16 sample (Figure 3J), despite the evident increase in surface roughness, the overall current signals remained very low. A few localized nanoscale spots showed slightly elevated conductivity (3.9 pA) compared to the surrounding matrix; however, no interconnected conductive networks were observed. The electrical signal levels were comparable to those of the pristine and PET‐5E14 samples, confirming that the inherent insulating properties of PET were largely retained.

**FIGURE 4:**
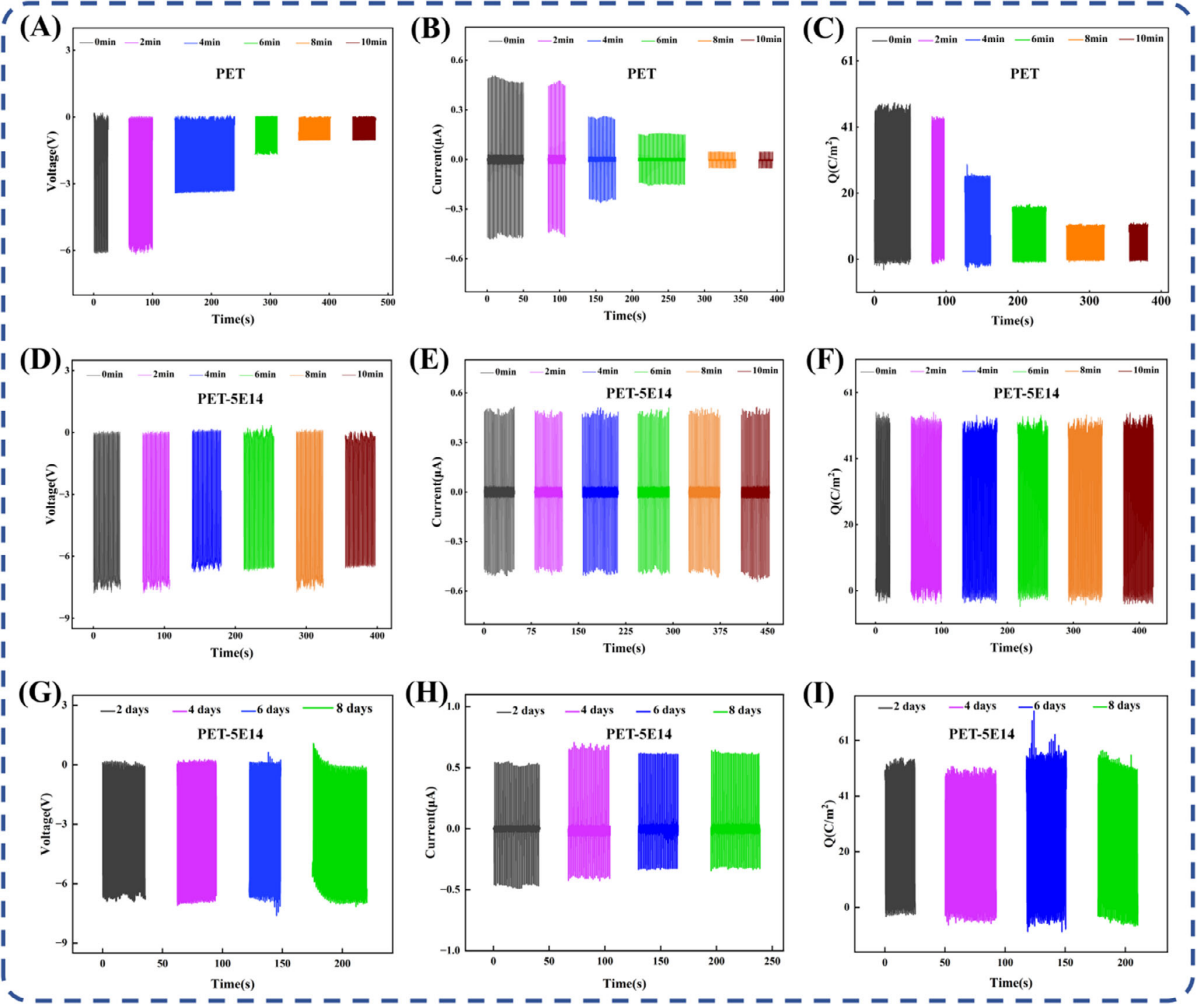
Output voltage (A), output current (B), and variation in surface charge (C) density of charge‐injected PET sample; output voltage (D), output current (E), and variation in surface charge density (F) of PET‐5E14 sample without additional charge injection measured over same duration and time intervals; long‐term stability test of PET‐5E14 sample, showing stable output voltage (G), output current (H), and surface charge density (I).

Charge injection resulting from ion irradiation is considered a secondary effect. Surface charges introduced during irradiation typically dissipate within several minutes under ambient conditions [38]. To differentiate these two effects, controlled neutralization and recharging experiments were designed. Two groups of samples, pristine PET and PET‐5E14, were selected for parallel comparative testing. For the pristine PET samples, artificial charge injection was carried out using a charge gun based on corona discharge, increasing the initial surface charge density to approximately 47 C/m^2^, which is comparable to that of the high‐performance modified samples. In contrast, the PET‐5E14 samples, irradiated with the optimal nitrogen ion dose (5 × 10^14^ ions/cm^2^), were tested without any additional charge injection and operated solely based on their intrinsically modified properties. Under identical environmental conditions with controlled temperature and humidity, TENG devices assembled with the two types of samples were continuously monitored. The surface charge density (Q), output voltage (V), and output current (I) were systematically measured and recorded at 2 min intervals. The results for the charge‐injected PET samples are illustrated in Figure 4A–C. After charge injection, the electrical output of pristine PET exhibits rapid and pronounced attenuation. Within approximately 8 min from the start of monitoring, the output voltage, current, and charge density decrease sharply and return to their original pre‐injection levels. In contrast, under the same monitoring duration and measurement frequency, the PET‐5E14 samples maintain consistently high and stable electrical outputs (Q, V, and I), as shown in Figure 4D–F, with no noticeable performance degradation. This behavior indicates that charges introduced through external injection are unstable and prone to dissipation, leading to only temporary and non‐intrinsic performance enhancement. Additional insulation testing was conducted using a digital multimeter (Figure S3), all samples maintained strong electrical insulation both before and after nitrogen ion implantation. These observations are consistent with findings from previous work [33]. To assess the durability of the modification effect, the PET‐5E14 sample underwent an eight‐day stability test under controlled ambient humidity conditions. In Figure 4G–I, the PET‐5E14 sample maintained highly stable electrical output throughout the entire testing period. The variations in output voltage, output current, and surface charge density all remained within ± 10% of their initial values, and no noticeable degradation was observed over time. The performance stability was Energy bandgap regulation, Flexible triboelectric polymers, Ion implantation, Polarity enhancement, Surface molecular modificationenhanced by more than 1440‐fold compared to pure charge injection.

### 3.3 Chemical Composition and Structural Characteristics

**FIGURE 5:**
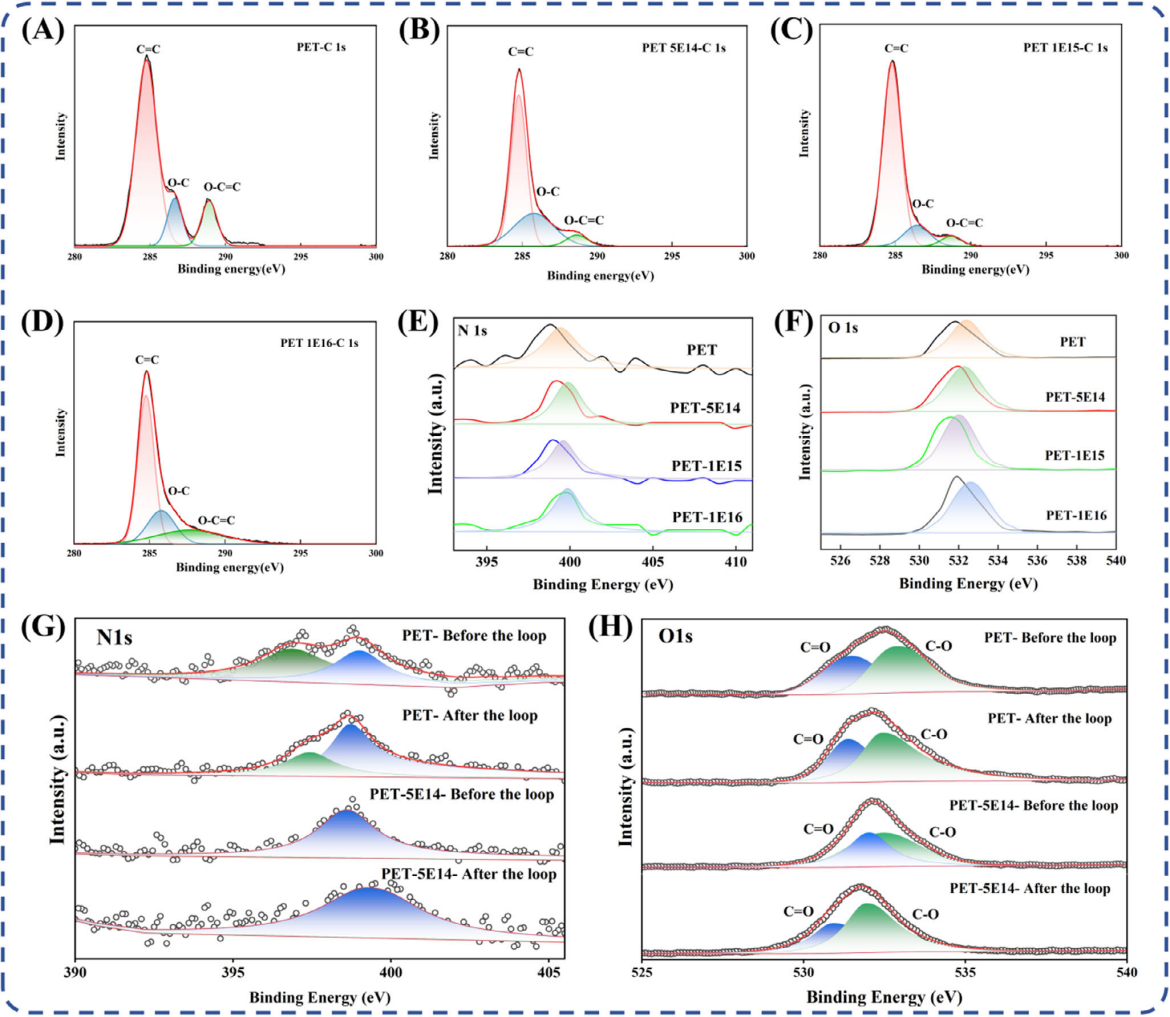
High‐resolution X‐ray Photoelectron Spectroscopy (XPS) spectra of PET samples implanted with the original condition and various doses of N ions. C1s spectra: Original sample (A); dose of 5E14(5 × 10^14^ ions/cm^2^)(B); dose of 1E15 (1 × 10^15^ ions/cm^2^) (C); and dose of 1E16 (1 × 10^16^ ions/cm^2^) (D); N 1s spectra for different doses (E); O 1s spectra at different doses (F); high‐resolution N 1s (G) and O 1s (H) spectra of PET‐5E14 and PET samples after cyclic testing compared with those obtained before cycling.

Figure 5 presents XPS and high‐resolution spectra of original and nitrogen‐ion irradiated PET samples. Full‐spectrum XPS (Figure S4A) analysis demonstrates that the nitrogen peak of the irradiated sample is significantly enhanced compared to the original sample. C 1s spectrum peak deconvolution with intensity comparison enables quantitative evaluation of chemical bond content changes and evolution patterns. Deconvolution of the C 1s spectrum identified three chemical bonds—C═C, C─O, and O─C═O—with quantitatively determined concentrations and binding energy shifts summarized in Table 1. Figure 5A–D show C 1s spectrum revealing dose‐dependent structural variations. At a low dose of 5E14, C═C bond content decreased to 60.6%, while C‐O bond content increased to 33.4%, accompanied by a binding energy shift from 286.6 to 285.8 eV. This can be attributed to the ester group's relatively lower bond energy (326 kJ/mol), which increases its susceptibility to nitrogen ion‐induced disruption, leading to the formation of C─O bonds and other structures. Under high‐dose irradiation, the contents of C─O and O─C═O bonds increased to 21.4% and 22.1%. This trend results from continuous energy input disrupting the molecular chain, promoting surface carbonization. Concurrently, C 1s spectrum anomalies suggest advanced carbonization stages with possible graphene‐like microdomains or amorphous carbon structures. This nonlinear variation in elemental concentration aligns with observations such as the reduced FTIR carbonyl peak intensity at 1720 cm^−1^. Figure 5E,F displays N 1s and O 1s spectra showing rising nitrogen content and declining oxygen content during irradiation. Altered C─O and O─C═O bond signals indicate potential nitrogen‐containing polar groups formation after N ions incorporation. The emergent N peak in the irradiated sample confirms successful nitrogen ions implantation, forming new polar bonds. This originates from nitrogen's high electronegativity (χ = 3.04), which promotes negatively polarized functional group generation through interactions with PET carbon atoms. After cyclic testing, the full‐spectrum XPS (Figure S4B), high‐resolution C 1s (Figure S4C), N 1s (Figure 5G), and O 1s (Figure 5H) spectra of the PET‐5E14 sample exhibit no noticeable peak shifts or significant changes in chemical states compared with those obtained before cycling. In particular, the N 1s signal, which serves as key evidence for successful nitrogen incorporation and is associated with nitrogen‐containing functional groups, maintains a stable binding energy after cyclic testing. As a control, the pristine PET sample also shows no substantial changes in its XPS survey spectrum after undergoing the same cyclic testing procedure.

**TABLE 1:** Variations in Chemical Group Concentrations and Binding Energies in pristine PET and implanted PET.

Sample	C═C [284.8 eV]	C─O [286.3 eV]	O─C═O [288.8 eV]
PET(reference)	66.3% (284.8 eV)	18.4% (286.3 eV)	15.3% (288.8 eV)
PET ‐ pristine	73.5% (284.8 eV)	13.3% (286.6 eV)	13.2% (288.9 eV)
PET‐5E14	60.6% (284.8 eV)	33.4% (285.8 eV)	6.0% (288.6 eV)
PET‐1E15	81.1% (284.8 eV)	13.1% (286.4 eV)	5.8% (288.7 eV)
PET‐1E16	56.5% (284.8 eV)	21.4% (285.8 eV)	22.1% (287.7 eV)

**FIGURE 6:**
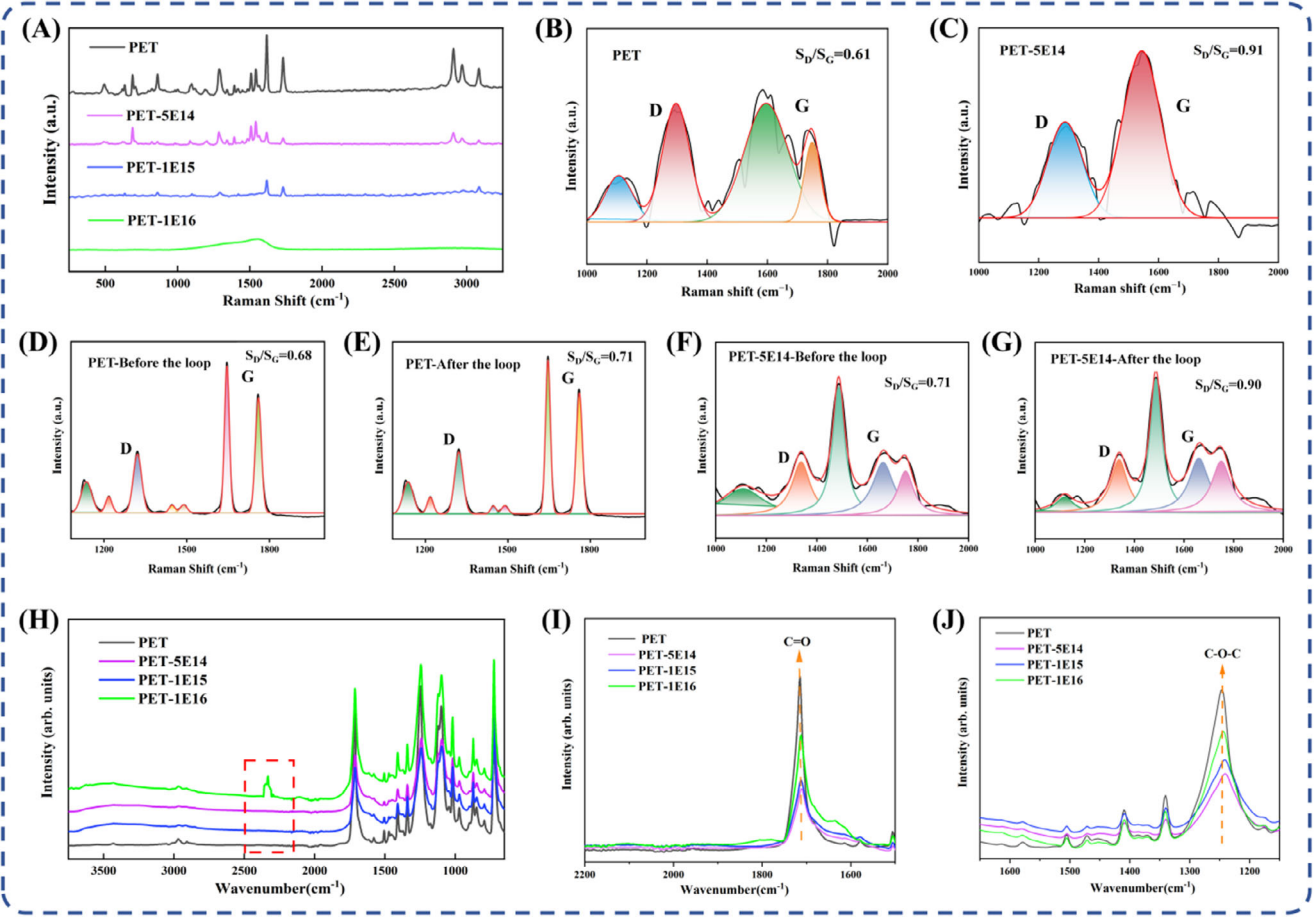
Raman spectra of the original PET samples and those implanted with different doses of N‐ions (A) and Raman Spectral Peak Chart (B,C), area ratios of D band to G band for PET before (D) and after (E) cycling, and for PET‐5E14 before (F) and after (G) cycling. The chemical structure of the original sample and the implanted sample of PET by ATR‐FTIR (H). Infrared spectra partial enlargement of (I,J).

Figure 6A presents Raman spectra of both pristine and N‐ion beam irradiated PET samples, revealing N‐ion beam irradiation effects on molecular structure and vibrational behavior. The pristine spectrum exhibits characteristic peaks: below 900 cm^−^^1^ (out‐of‐plane C–H bending of benzene rings), 1289 cm^−1^ (benzene ring stretching), 1509 cm^−1^/1542 cm^−1^ (symmetric stretching of para‐substituted benzene rings), 1730 cm^−1^ (ester C = O stretching), 2908 cm^−1^/2968 cm^−1^ (CH_2_ antisymmetric stretching), and 3077 cm^−1^ (aromatic C‐H stretching). Post‐irradiation Raman spectrum exhibits significant attenuation of characteristic peaks. At 5 × 10^14^ ions/cm^2^, the 1730 cm^−1^ ester group peak shows marked intensity reduction. This is attributed to nitrogen incorporation suppressing symmetric vibrational modes of the electric dipole moment, thereby diminishing Raman activity at these spectral positions. Meanwhile, N‐ion irradiation partially alters PET's chemical structure by cleaving ester groups, C─H bonds, and benzene ring substituents, while maintaining benzene ring stability with minimal disruption to its extended *π*‐bond system. This is evidenced by Raman spectroscopy showing only slight intensity reduction at 1617 cm^−1^, consistent with the XPS findings. At 1 × 10^15^ ions/cm^2^, the spectral intensity decreases moderately yet remains above unirradiated levels. This anomaly is attributed to partial carbonization and cross‐linking under high‐dose irradiation, reducing molecular vibrational activity. At 1 × 10^16^ ions/cm^2^, the spectral intensity was markedly reduced with concurrently smoothed peak profiles. This indicates that high‐dose irradiation promoted carbonized and disordered structures formation, thereby suppressing molecular vibrations. The intensity ratio of the D band (∼1350 cm^−1^) to G band (∼ 1580 cm^−1^) (S_D_/S_G_) serves as a key metric for assessing structural disorder in carbon‐based materials and shifts in sp^3^/sp^2^ hybridization states. Raman spectral deconvolution was conducted for all samples, with results presented in Figure 6B,C and Figure S5A,B. Unirradiated PET samples showed a low S_D_/S_G_ ratio of 0.61, with weak D and G band intensities, indicative of ordered aromatic rings and ester bonds with minimal carbon disorder. At 5 × 10^14^ ions/cm^2^, irradiation induced progressive intensity reduction of Raman features, culminating in broadened D and G bands. The S_D_/S_G_ ratio increased to 0.91, with marked D‐band enhancement and moderate G‐band intensification, reflecting C─C bond cleavage and increased unsaturated bond density (e.g., C≡N, C = C, and C = N). This indicates that low‐dose N ion beam irradiation induced surface molecular chain scission, generating amorphous carbon phases and defects while increasing material polarity. With elevated doses, S_D_/S_G_ reached 1.05 (Figure S5A), indicating increased unsaturated bond concentration, enhanced defect density, and additional sp^2^‐hybridized carbon formation. At 1 × 10^16^ ions/cm^2^, the D band nearly vanished (Figure S5B), likely due to extensive PET chain scission and carbonization from high‐dose N^+^ implantation. This caused severe structural disruption and substantial sp^2^‐carbon formation. In Figure S5C, the full‐range Raman spectra of PET and PET‐5E14 remain essentially unchanged before and after cycling, indicating the inherent mechanical stability of the PET polymer film. For the PET sample, the Raman spectra recorded before and after cyclic testing exhibit a high degree of overlap, and the S_D_/S_G_ ratio also remains essentially unchanged following cycling (Figure 6D,E). For the PET‐5E14 sample, the Raman spectra recorded before and after cyclic testing exhibit a high degree of overlap. Characteristic peaks, including the ester C = O stretching vibration (∼1730 cm^−^^1^) and aromatic ring vibration modes, show no noticeable shifts in peak position or relative intensity. Moreover, the intensity ratio of the D band to the G band (S_D_/S_G_), which is commonly used to evaluate irradiation‐induced defects and the degree of carbon structural disorder, shows no significant variation before and after cyclic testing (Figure 6F,G). These results suggest that the specific defect states and microstructural features introduced by low‐dose nitrogen ion implantation, such as the increased fraction of sp^2^‐hybridized carbon, possess good mechanical and environmental stability and are not significantly affected by prolonged friction cycles.

FTIR spectroscopy confirmed N‐ion beam irradiation‐induced molecular structural changes in PET through absorption peak variations. Figure 6H compares unirradiated and irradiated PET spectra, with magnified views provided in Figure 6I,J. Unirradiated PET shows characteristic peaks: 1240–1270 cm^−1^ (ester group stretching), 1720 cm^−1^ (C = O stretching), and ∼3000 cm^−1^ (C‐H stretching in aliphatic chains). The prevalence of these ester‐dominated peaks confirms their role as the material's primary functional components. Post‐irradiation spectra maintain identical peak positions with stable relative intensities, consistent with established literature. Increasing irradiation dose induced progressive changes. At 5 × 10^14^ ions/cm^2^, decreased intensities at 1715 cm^−1^ (C═O) and 1250 cm^−1^ (C─O─C) indicate certain ester bond cleavage. Concurrently, free radical reactions disrupt aromatic C═C bonds, generating small molecular fragments that reduce C‐H content. At 1 × 10^15^ ions/cm^2^, overall spectral intensity declines significantly. At 1 × 10^15^ ions/cm^2^, overall spectral intensity declines significantly. At 1 × 10^16^ ions/cm^2^, further molecular chain degradation reduces intensity and reveals a distinct new peak at 2330 cm^−1^ (Figure 6H)‐absent at other doses—‐indicating specific bond cleavage that may impede charge transfer. Such specific bond rupture may drive the deterioration of electrical properties relative to unirradiated samples with increasing irradiation dose. The robust correlation between the surface‐sensitive XPS and bulk‐phase Raman/FTIR detection confirms both spatial uniformity and structural consistency of the modification effects. We found that the core regulatory mechanisms of ion implantation vary depending on the dose administered (Figure S6). Furthermore, these findings elucidate the progressive regulatory mechanism induced by ion implantation: ester group cleavage → bond reconstruction → surface carbonization.

### 3.4 Functional Verification and Analysis

**FIGURE 7:**
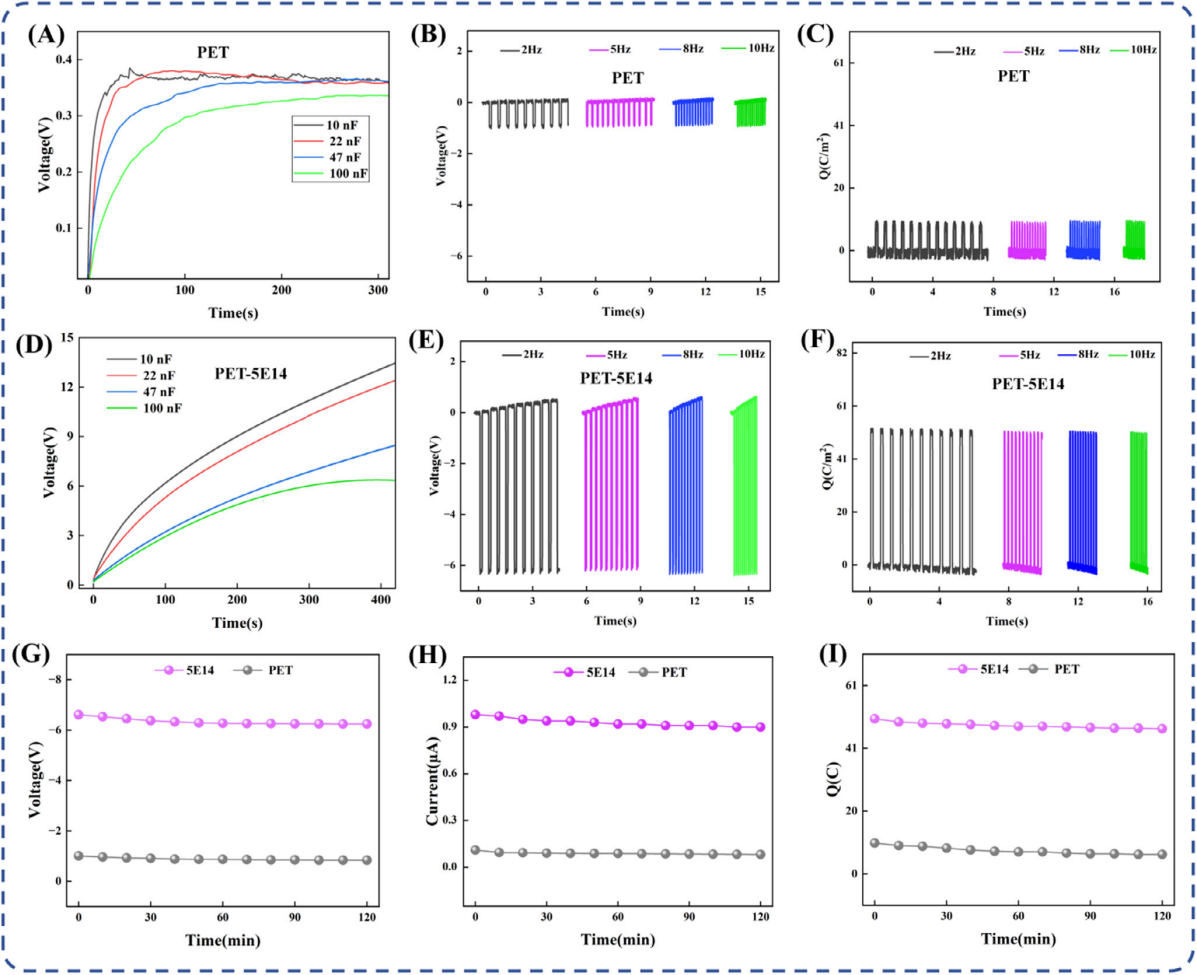
The charging curve of PET‐TENG (A) and PET‐5E14‐TENG (D); frequency test results of PET‐TENG (B,C) and PET‐5E14‐TENG (E,F), and durability test results (G–I) of PET‐5E14‐TENG and PET‐TENG.

To evaluate the practical direct current (DC) energy harvesting capability of the TENG, this study conducted capacitor charging tests under DC operating conditions. The TENG device fabricated from PET material achieved a charging voltage of only 0.34 V (Figure 7A), in contrast, the TENG constructed using PET‐5E14 material exhibited significantly enhanced performance, charging a 100 nF capacitor to approximately 6.13 V (Figure 7D)‐a more than 18‐fold increase. The research further calculated the mechanical‐to‐electrical energy conversion efficiency η by accurately measuring the electrical energy output and mechanical energy input over a complete operating cycle, following established methodologies [39, 40]. The results indicate that the TENG assembled with optimally irradiated PET films exhibits a 372.7‐fold improvement in conversion efficiency compared to the unmodified PET‐based device. These findings confirm that nitrogen ion implantation substantially enhances the energy conversion capability of PET, underscoring its potential as a high‐performance triboelectric material (detailed calculation procedures are provided in Section 7).

Response speed plays a critical role in determining whether a TENG can accurately capture rapidly changing mechanical signals. To assess this characteristic, this study evaluated the output performance of both the optimally irradiated PET‐5E14 TENG and the pristine PET‐based device across a wide frequency range from 2 to 10 Hz. Notably, this range encompasses the fundamental frequencies associated with most periodic human physiological activities, including joint motion at varying intensities (1–5 Hz) and heart rate monitoring (1–1.67 Hz) [41, 42]. The PET‐TENG exhibited relatively low output voltage (Figure 7B), current (Figure S7A), and surface charge density (Figure 7C) across the entire testing range. In contrast, the PET‐5E14‐TENG demonstrated superior performance in terms of voltage (Figure 7E), current (Figure S7B), and surface charge (Figure 7F) density over the same testing range, significantly outperforming the pristine PET‐TENG. Moreover, no signal attenuation was observed even at 1 Hz, indicating that the irradiation‐modified device sustains high responsiveness not only to subtle physiological signals but also to the high‐frequency mechanical stimuli generated during vigorous physical movement. In practical applications, long‐term mechanical durability is equally essential. To verify this, a cyclic operational stability test was conducted. In Figure 7G–I, the PET‐5E14‐TENG was subjected to vertical contact–separation cycles at 5 Hz for 120 min (36 000 cycles). Throughout the test, no significant degradation in output voltage or current was detected, confirming the device's excellent durability. This remarkable stability can be attributed to two main factors. First, as evidenced by the AFM analysis in Figure 3F, ion implantation at the optimal dose (5 × 10^14^ ions/cm^2^) resulted in only discrete nanoscale protrusions on the PET surface, preserving the overall structural integrity and preventing mechanical failure due to collapse. In our work, the ion implantation introduced highly polar covalent bonds, such as C≡N and C = N. Combined with the SRIM simulation results, since the modification and existence range of N ion implantation is from 0 to 300 nm with a certain depth, surface wear cannot significantly affect the modification effect, and the wear resistance of the material's performance is significantly improved. This modification method helps maintain a consistent surface charge density over time. The combination of rapid dynamic responsiveness and long‐term mechanical endurance highlights the great potential of this atomically precise modification approach in developing high‐performance triboelectric materials, offering a solid foundation for their integration into wearable electronics and health monitoring technologies.

## 4 Conclusion

In summary, through systematic irradiation of flexible PET materials with graded nitrogen ion doses combined with spectral and electrical characterization, we elucidated the regulatory pathway for surface molecular structure modulation in flexible polymers: ester bond cleavage followed by bond reconstruction and subsequent surface carbonization. DFT calculations enabled microscopic electronic structure analysis of bandgap structure and ESP variations. This method validated a synergistic regulation mechanism characterized by bandgap narrowing, surface ESP redistribution, and polarity enhancement, collectively elucidating the enhancement for surface charge transport. Collectively, these effects significantly improved surface charge transport capabilities. Macroscopic electrical testing confirmed 13‐, 10‐, and 7‐fold enhancements in TENG output voltage, current, and surface charge density, respectively, versus pristine PET. With stability exceeding 1440‐fold that of pure charge injection. Charge injection experiments conducted on pristine PET samples revealed rapid dissipation of the injected charges within a short duration. In contrast, the irradiated PET‐5E14 sample exhibited exceptional stability, surpassing that of the pristine PET by a factor of 1440. XPS and Raman spectroscopy analyses before and after cycling tests indicated no significant alterations in the chemical structure of the PET‐5E14 sample. These findings confirm that the proposed approach constitutes a precise and stable strategy for atomic‐scale modification. The irradiated sample exhibits a 372.7‐fold enhancement in energy conversion efficiency and demonstrates high sensitivity and outstanding wear resistance when fabricated into a device, showcasing significant potential for the development of high‐performance triboelectric materials.

DFT calculations enabled microscopic electronic structure analysis of bandgap structure and ESP variations. This method validated a synergistic regulation mechanism characterized by bandgap narrowing, surface ESP redistribution, and polarity enhancement, collectively elucidating the enhancement for surface charge transport. Specifically, HOMO‐LUMO calculations quantified a 1.337 eV bandgap contraction after low‐dose implantation, which facilitated electron transitions to the conduction band, and improved carrier migration efficiency. Concurrently, the electronic energy of the molecular structure on the PET surface increases by approximately 4.3 Hartree due to the addition of nitrogen‐induced polar groups, signaling surface charge redistribution with increased localized negative charge and enhanced the polarity of the PET molecule. Collectively, these effects significantly improved surface charge transport capabilities. Macroscopic electrical testing confirmed 13‐, 10‐, and 7‐fold enhancements in TENG output voltage, current, and surface charge density, respectively, versus pristine PET. This enhancement was predominantly attributed to low‐dose N‐ion implantation effects, where introduced N ions simultaneously increase the molecular chain dipole moments and enhance electronegativity. Additionally, the high electronegativity and chemical reactivity of N ions enable the formation of new polar functional groups through reactions with PET carbon, further boosting the material's electronegativity.

This study addresses the core scientific question of atomic‐scale structural units and their assembly mechanisms by integrating ion implantation technology—originally developed in nuclear physics research—with flexible intelligent polymers. Through this interdisciplinary approach, we have enabled on‐demand customization of surface functionalities and interfacial structures in polymer‐based flexible materials, thus establishing a novel strategy for atomic‐level manufacturing. Our findings elucidate the precise surface modification mechanism of polymer functional groups induced by ion implantation. Beyond enhancing triboelectric performance, this new strategy also enables precise control over surface wettability by tailoring hydrophobic functional groups. The enhancement of material hydrophobicity can be used to develop waterproof and corrosion‐resistant materials for wearable devices. In addition, this surface engineering method can be extended to modify the spatial network structure of flexible materials via crosslinking, thereby improving the compactness of the material. Such improvements are especially valuable for biomedical applications, such as enhancing the compatibility and stability of flexible functional materials. Altogether, this ion implantation‐based strategy offers a new pathway for the fabrication of high‐performance wearable sensors and advanced flexible functional materials.

## Conflicts of Interest

The authors declare no conflicts of interest.

## Supporting information




**Supporting File**: advs73910‐sup‐0001‐SuppMat.docx.

## Data Availability

The data that support the findings of this study are available from the corresponding author upon reasonable request.
