# Supplementary material for: Gradient Ion Beams Regulate Surface Group Modification to Enhance Interfacial Charge Transport in Triboelectric Polymers
Source: Adv Sci (Weinh). 2026 Jan 21;13(18):e18257. doi: 10.1002/advs.202518257 (PMC13042401; doi:10.1002/advs.202518257)
Supplement: Supplementary file 1 — Supporting File: advs73910‐sup‐0001‐SuppMat.docx. [file ADVS-13-e18257-s001.docx]

Supporting Information

Gradient Ion Beams Regulate Surface Group Modification to Enhance Interfacial Charge Transport in Triboelectric Polymers

*Yi Chen^✝,1 , 3^,* *Yuliang Yao^✝，2^, Xinya Shen ^1, 3^, Meiling Gong ^1,3^, Chuan Xu ^2^, Chunliang Zhou ^1, 3^, Fuqiu Ma ^1, 3^, Jian Zhang ^4^, Xiangyu Chen ^5^, Yanxia Liang ^6^, Engang Fu ^2,^ *, Yong Fan ^1, 2, 3,^ **

^1^ YanTai research institute, Harbin Engineering University, Yantai 265503, China

^2^ State Key Laboratory of Nuclear Physics and Technology, School of Physics, Peking University，Beijing 100871, China

^3^ College of Materials Science and Chemical Engineering, Harbin Engineering University, Harbin 150001, China

^4^ College of Energy, Xiamen University, Xiamen 361005, China

^5^ CAS Center for Excellence in Nanoscience, Beijing Key Laboratory of Micro-nano Energy and Sensor, Beijing Institute of Nanoenergy and Nanosystems, Chinese Academy of Sciences, Beijing 100083, China

^6^ State Key Laboratory of Chemistry for NBC Hazards Protection, Beijing 102205, China

^✝^Equal contribution

* Corresponding author.

E-*mail address*: Engang Fu, efu@pku.edu.cn

Yong Fan, yfan1@hrbeu.edu.cn

Supporting Text

**Section 1. The process to calculate and simulate the ion implantation by The Monte Carlo**

The Monte Carlo method is used to calculate and simulate the ion implantation process, and further analyze the material structure changes and ion motion trajectories during this period.^1^ This calculation method can track the transmission and collision of a large number of incident particles in the medium. Through the stay and movement range of the ions in the substance, the energy loss and various parameters of the secondary particles can be calculated during the entire implantation process. The distribution of displacements per atom (DPA) along depth can be expressed as:

$$DPA\left( x \right)=\frac{FSv\left( x \right)\Delta d}{NS\Delta d}=\frac{Fv\left( x \right)}{N} \left( 1 \right)$$

where *F* is the implanted ion flux (unit is ions/cm^2^), *S* is the surface area of the target material, *x* is the incident depth, *∆d* is the material thickness, *N* is the atomic density of the material (unit is atoms cm^-3^), and *v(x)* represents the average number of displaced atoms per ion (unit thickness is per angstrom) at depth of *x*. The damage along the depth during the ion implantation process shows in Figure S1. The concentration distribution of implanted ions using the following equations:

$$Concentration=\frac{N_{i}}{N}=\frac{Fu\left( x \right)}{N} \left( 2 \right)$$

where *N_i_* is the concentration of implanted ions, *u(x)* represents the average probability that each ion exists in the unit thickness at depth *x*.


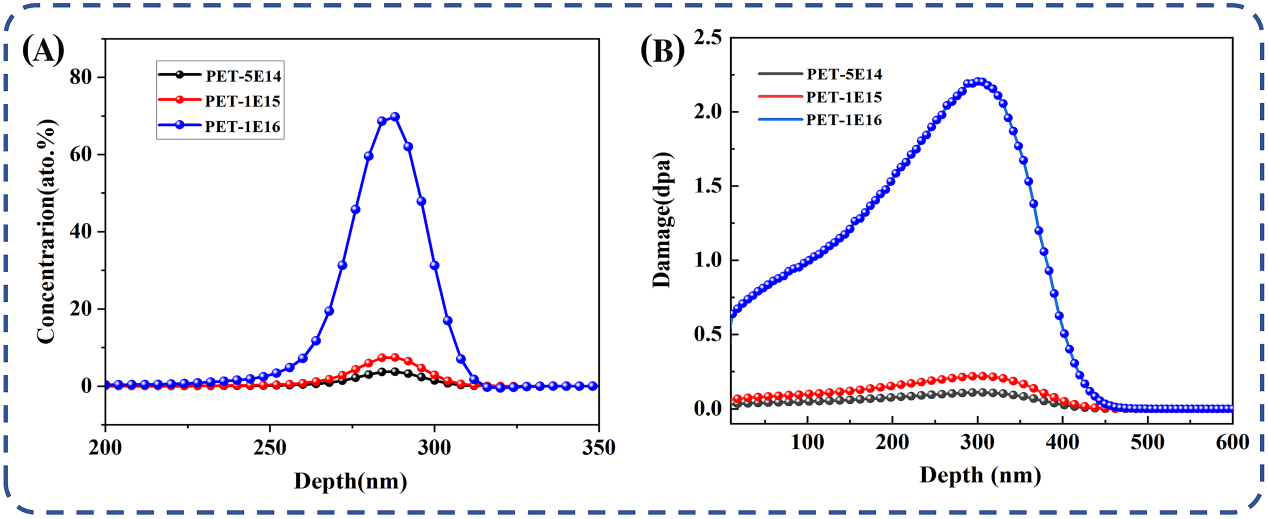


**Figure S1.** Radiation damage (DPA) (A) and concentration of the injected N ion along the depth in PET (B) by N ion implantation at various dose Levels according to SRIM.

**Section 2.Scanning electron microscope ( SEM )**


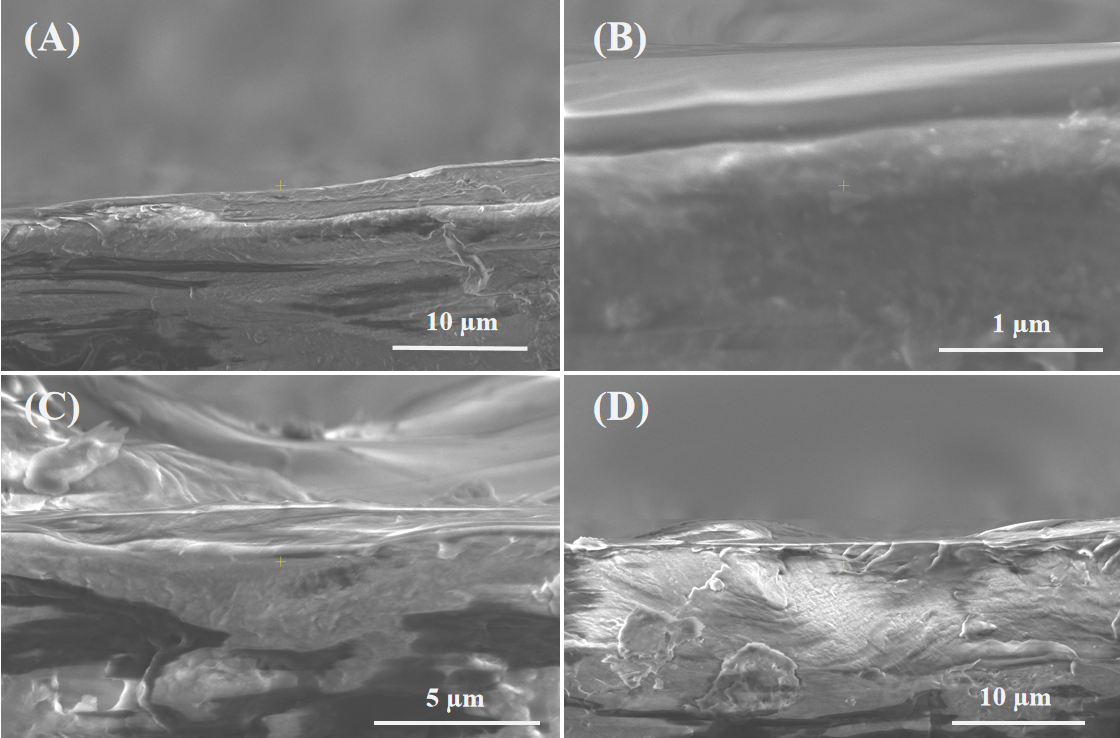


**Figure S2.** SEM images of the original PET sample (A-B), and high-dose implantation PET-1E16 (C-D).

**Section 3. Insulation test**


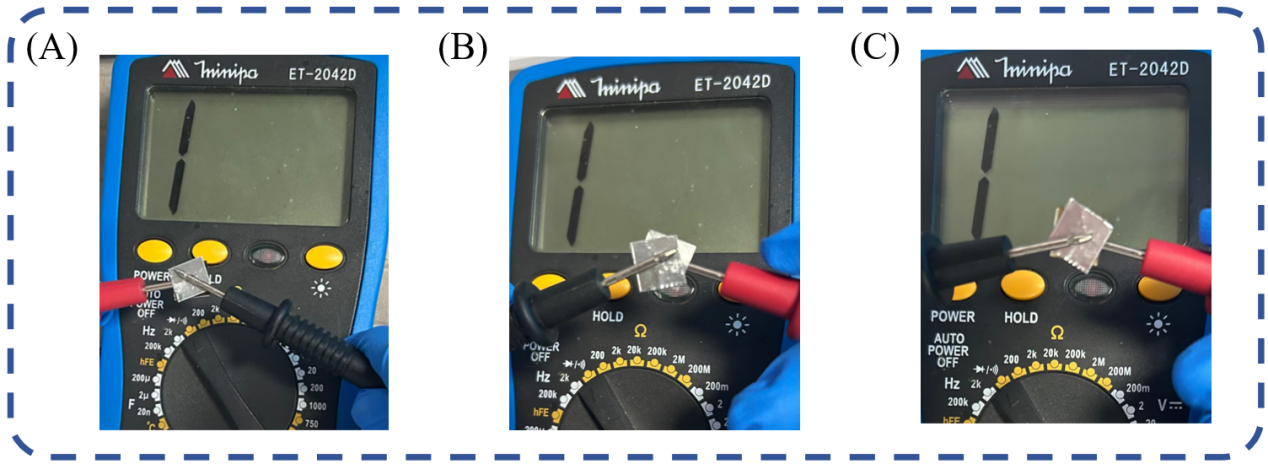


**Figure S3.** The Al electrode is attached to the back of the polymer film and detect between the electrode and the polymer with two probes of the multimeter, showing no electrical signal. PET (A) ; PET-5E14 (B) and PET-1E16 (C).

**Section 4. XPS spectrum graph**


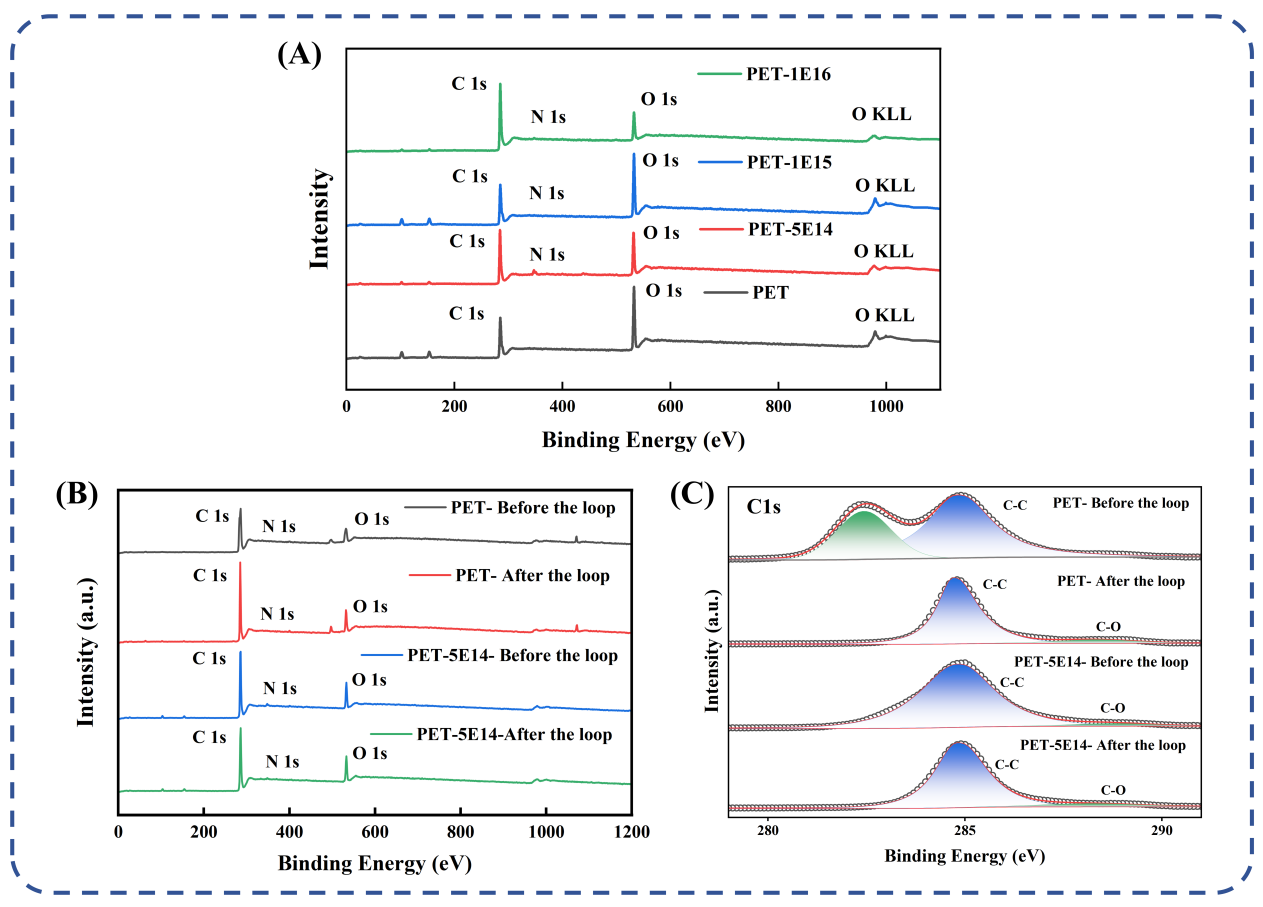


**Figure S4.** Full XPS spectra of PET samples prior to and following implantation (A); full-spectrum XPS (B) and high-resolution C 1s (C) spectra of PET-5E14 and PET samples after cyclic testing compared with those obtained before cycling.

**Section 5. Raman spectrum graph**


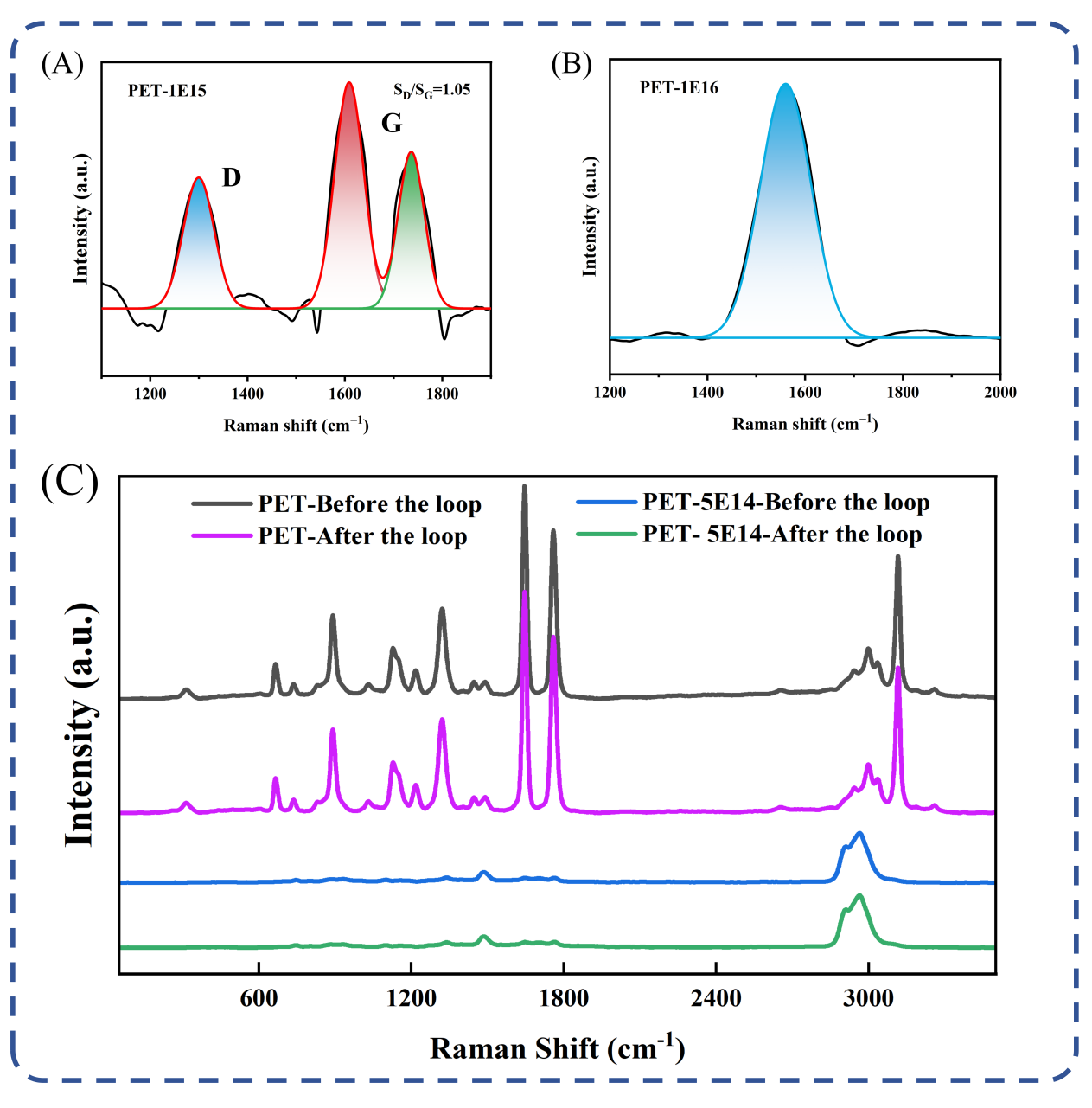


**Figure S5.** Raman spectra of PET samples under varying doses of N-ion implantation and the corresponding D/G peak area ratios (A) 1E15 and (B) 1E16, the full-range Raman spectra (C) of PET and PET-5E14 remain essentially unchanged before and after cycling. Owing to prolonged sample storage, the characteristic Raman peaks have broadened, the background intensity has increased, and the number of impurity peaks has increased.

**Section 6. Core regulatory mechanisms**


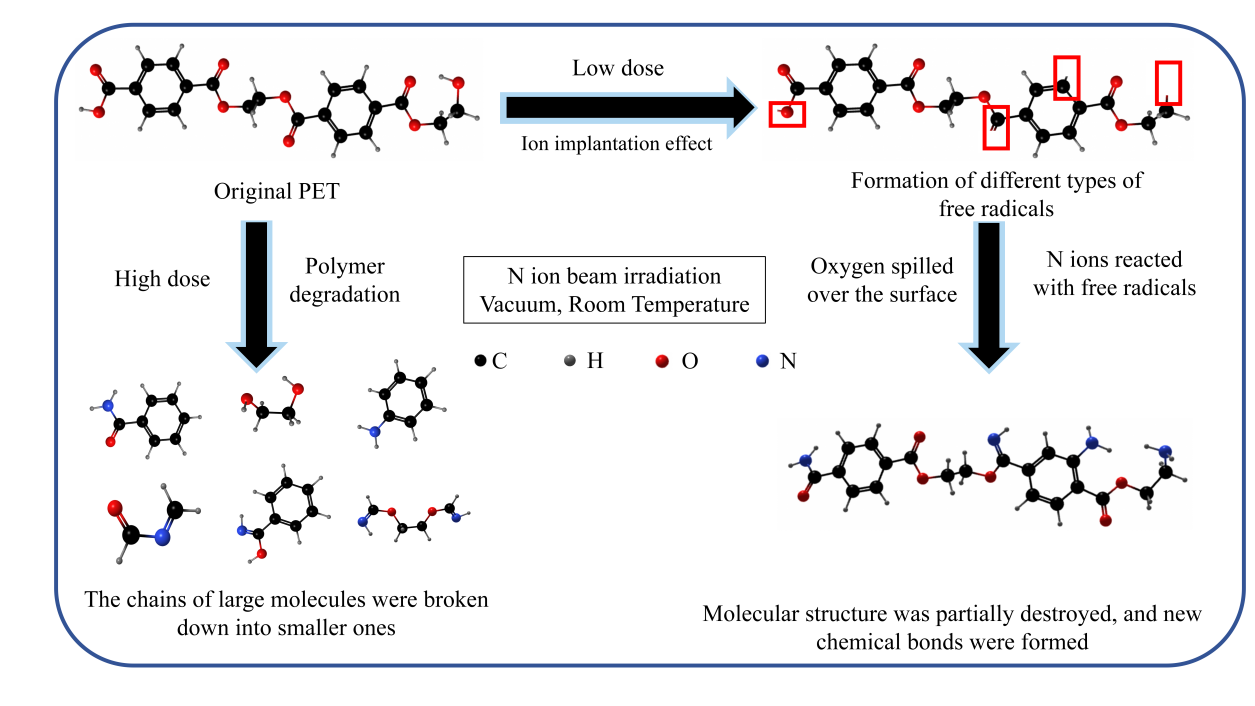


**Figure S6.** Schematic illustration of the regulatory mechanism under varying doses of ion implantation.

**Section 7. Calculation for energy conversion efficiency**

Energy conversion efficiency, $\text{η}$ of a TENG may be defined as：

$$\eta=\frac{E_{electrical}}{E_{mech}}\times100\%=\frac{\frac{1}{2}CV^{2}}{E_{mech}}\times100\%$$

Among these parameters, E_electrical_ and E_mechanical_ denote the output electrical energy and input mechanical energy, respectively. C represents the capacitance value, while V denotes the voltage across the capacitor. Accordingly, the ratio of the energy conversion efficiency η₁ of PET-5E14-TENG to the energy conversion efficiency η₂ of PET-TENG is derived as：

$$\frac{\text{η}_{\text{1}}}{\text{η}_{\text{2}}}\text{=}\frac{\frac{\text{1}}{\text{2}}\text{×47×}\text{10}^{\text{-9}}\text{×}\text{9.55}^{\text{2}}}{\frac{\text{1}}{\text{2}}\text{×100×}\text{10}^{\text{-9}}\text{×0.}\text{33}^{\text{2}}}\text{=372.7}$$

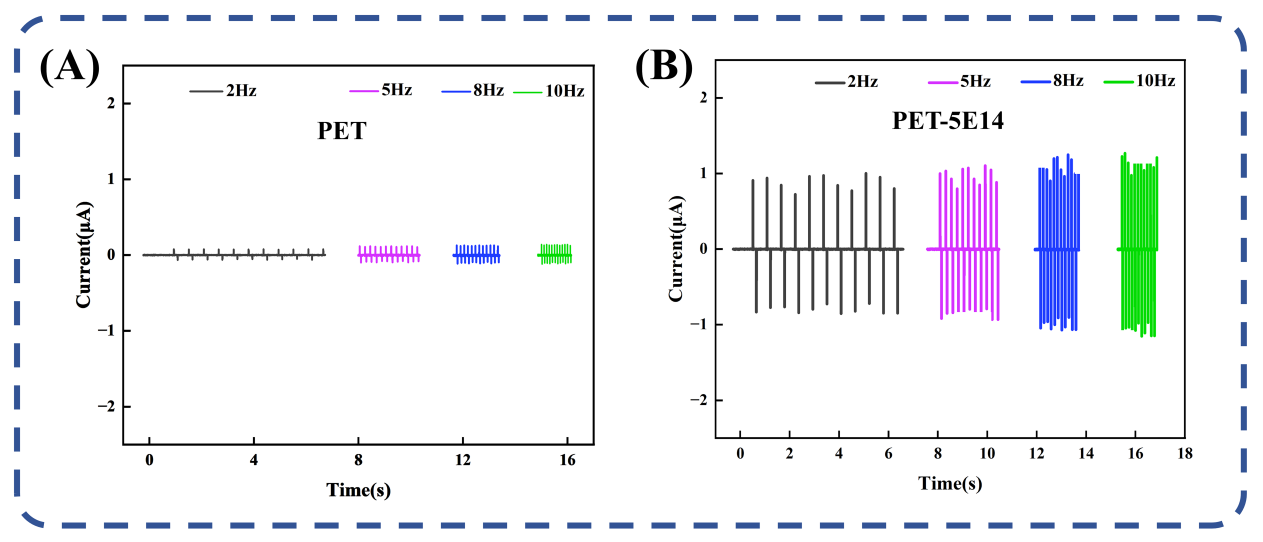


**Figure S7.** The test results of current frequency for PET-TENG (A) and PET-5E14TENG (B).
